# Supplementary material for: Red GaPAs/GaP Nanowire-Based Flexible Light-Emitting Diodes
Source: Nanomaterials (Basel). 2021 Sep 29;11(10):2549. doi: 10.3390/nano11102549 (PMC8538214; doi:10.3390/nano11102549)
Supplement: Supplementary file 1 [file nanomaterials-11-02549-s001.zip › nanomaterials-1369225-supplementary.pdf]

Supporting Information

Red GaPAs/GaP Nanowire-Based Flexible Light-Emitting Diodes

Vladimir Neplokh [1,2,*], Vladimir Fedorov [1,2], Alexey Mozharov [2], Fedor Kochetkov [2], Konstantin Shugurov [2],
Eduard Moiseev [2,3], Nuño Amador-Mendez [4], Tatiana Statsenko [5,6], Sofia Morozova [5,6], Dmitry Krasnikov [7],
Albert G. Nasibulin [7,8], Regina Islamova [9], George Cirlin [2], Maria Tchernycheva [4] and Ivan Mukhin [1,2]

1 High School of Engineering Physics, Peter the Great St. Petersburg Polytechnic University,
Polytechnicheskaya 29, 195251 St. Petersburg, Russia; vfedorov.fl@mail.ioffe.ru (V.F.); imukhin@yandex.ru (I.M.)
2 Department of Physics, Alferov University, Khlopina 8/3, 194021 St. Petersburg, Russia; mozharov@spbau.ru (A.M.); azemerat@rambler.ru (F.K.); shugurov17@mail.ru (K.S.); moiseev@spbau.com (E.M.); cirlin@beam.ioffe.ru (G.C.)
3 Laboratory of quantum optoelectronics, National Research University Higher School of Economics,
Kantemirovskaya 3A, 194100 St. Petersburg, Russia
4 Centre of Nanosciences and Nanotechnologies, UMR 9001 CNRS, University Paris-Saclay, 10 Boulevard Thomas Gobert, 91120 Palaiseau, France; nuno.amador@c2n.upsaclay.fr (N.A.-M.); maria.tchernycheva@u-psud.fr (M.T.)
5 Department of Chemistry, ITMO University, Lomonosova 9, 197101 St. Petersburg, Russia; statsenko@scamt-itmo.ru (T.S.); sofiionova@yandex.ru (S.M.)
6 N.E. Bauman Moscow State Technical University, 2nd Baumanskaya str. 5/1, 105005 Moscow, Russia
7 Skolkovo Institute of Science and Technology, Bolshoy Boulevard 30/1, 121205 Moscow, Russia;
d.krasnikov@skoltech.ru (D.K.); A.Nasibulin@skoltech.ru (A.G.N.)
8 Department of Chemistry and Materials Science, Aalto University, FI-00076 Espoo, Finland
9 Institute of Chemistry, Saint Petersburg State University, Universitetskaya Emb. 7/9, 199034 St. Petersburg, Russia; r.islamova@spbu.ru
* Correspondence: vneplox@gmail.com

Section 1 Numerical modeling

Modeling was performed for the fixed doping level of the emitters and the active region, 10^{18} and 10^{15} cm^{-3}, respectively.

The bandgap value, direct/indirect bandgap transition point, and work function depending on material composition were defined using [1]. As it is reproduced from [1] in Figure S1a, the increase of P content leads to a rise of the bandgap value for all three subbands of the conduction band. Meanwhile, at a P content of 0.48 the minimal conduction band energy level shifts from the Γ- to X-valley, and the GaPAs material becomes indirect. The subband shift is accompanied with a dramatic change of the effective electronic state density in the conduction band due to the different effective electron mass value in the X-valley. Calculation of the effective electronic state density was based on the effective masses for valence and conduction subbands presented in Table S1. The calculated effective state density vs. P content x in GaP_xAs_{1-x} material is presented in Figure S1b.

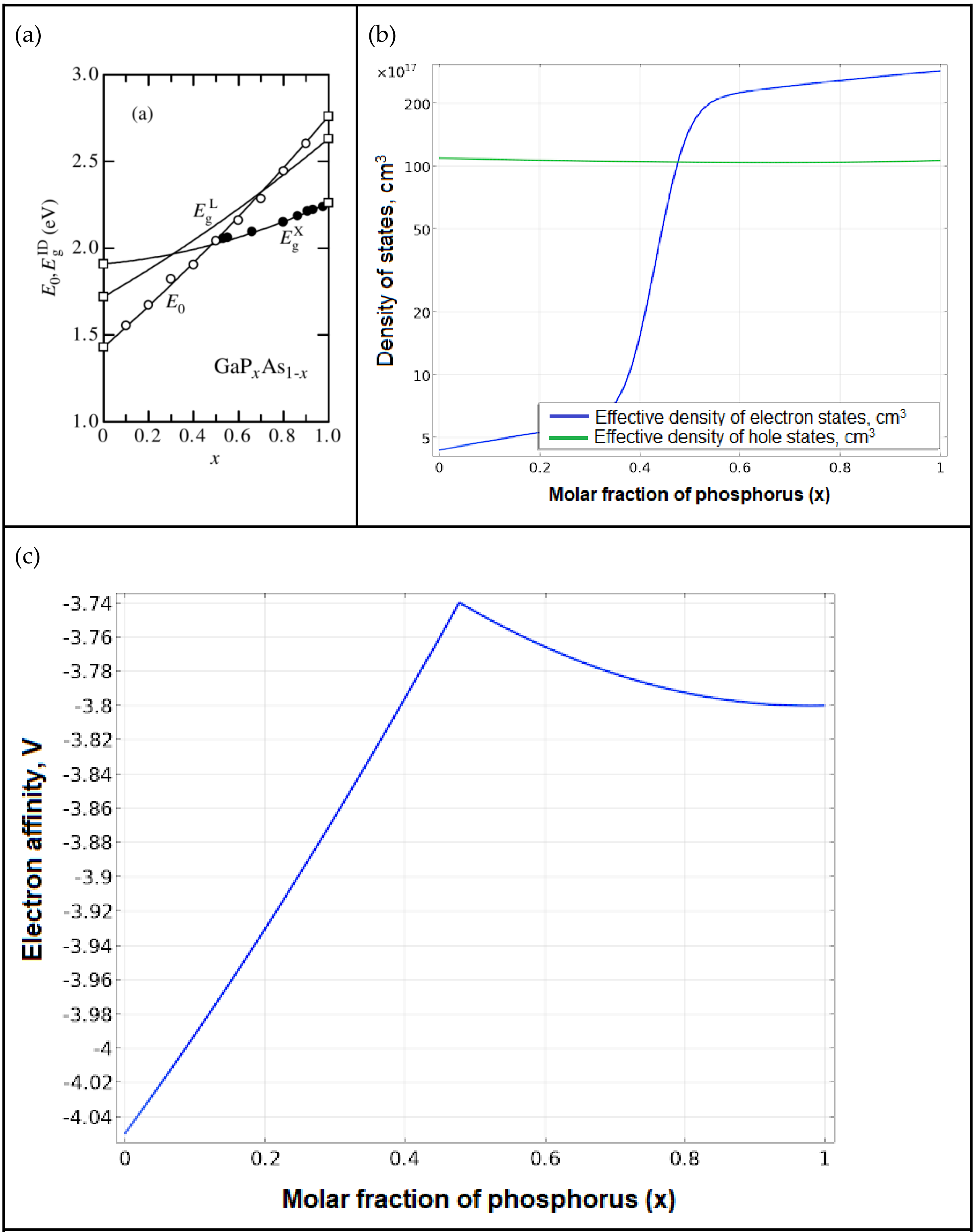

Figure S1. (a) Band gap of the conductive subbands (reproduced from [1]), calculated (b) effective electronic (blue line) and hole (green line) state density, and (c) electron affinity for different P content x in the GaP_xAs_{1-x} compound.

Table S1. Electron and hole effective masses depending on the valley and direction.							
	Electron effective mass			Hole effective mass			
	m_Γ	m_X^l	m_X^t	$m_{hh}^{[100]}$	$m_{hh}^{[111]}$	$m_{lh}^{[100]}$	$m_{lh}^{[111]}$
GaAs	0.067	1.3	0.23	0.33	0.78	0.09	0.077
GaP	0.114	6.9	0.252	0.34	0.66	0.2	0.15

The literature data study did not provide information about the dependence of the electron affinity χ for the modeled GaP_xAs_{1-x} compounds, so we used the data for pure GaAs and GaP materials with χ = 4.05 and 3.8 eV, respectively. The valence band of both GaAs and GaP is formed by Ga atom electronic orbitals having the base energy level in the Γ point that preserves its configuration in the GaP_xAs_{1-x} compound. Therefore, it can be used to approximate the electron affinity via a linear approximation of the valence band edge position related to the vacuum level (χ + E_G) and the bandgap value dependency on the compound content (Figure S1a). The calculated electron affinity vs GaP_xAs_{1-x} compound content is presented in the Figure S1c.

The carrier mobility is inversely proportional to the carrier effective mass in the studied subband. The electron and hole mobility was calculated using the values in the Table S1 via an estimation of the conduction effective mass dependency on the content. Herewith, the conductive electron effective masses were calculated independently for the Γ- and X-subbands. The calculation was carried out under an assumption that the Γ-valley electron scattering process is different to the X-valley. Another assumption was that the momentum dissipation time in both valleys is constant for all compound contents, so the pure GaAs and GaP mobility values can be used. Thus, the calculation of the functional dependency of the mobility vs. content was performed by taking into account the scattering and dissipation mechanism and the probability of electrons to be found in both valleys, i.e.,

$$\frac{1}{\mu_e} = \frac{1}{\mu_{GaAs}} \cdot \frac{m^{\Gamma}_{cond}(x)}{m^{\Gamma}_{cond\,GaAs}} \cdot F(\Delta E) + \frac{1}{\mu_{GaP}} \cdot \frac{m^{X}_{cond}(x)}{m^{X}_{cond\,GaP}} \cdot F(-\Delta E),$$

where $\mu_{GaAs,GaP}$ is the electron mobility in the GaAs or GaP material at a given doping level, respectively, m_{cond} is the effective mass in a respective conduction subband, F is the Fermi function on ΔE which is the difference between the minimal energy level in Γ- and X-subbands at the given content. The values calculated by the presented formula are in a good agreement with the experimental data [1].

Meanwhile, the hole mobility was calculated using a simple linear approximation of the reciprocal mobilities in pure GaAs and GaP:

$$\frac{1}{\mu_h} = \frac{1-x}{\mu_{GaAs}} + \frac{x}{\mu_{GaP}},$$

where μ $_{GaAs,GaP}$ is the hole mobility in pure GaAs or GaP material at a given doping level, respectively, and x is the P content in the compound. The derived mobility vs. the content dependency is presented in Figure S2.

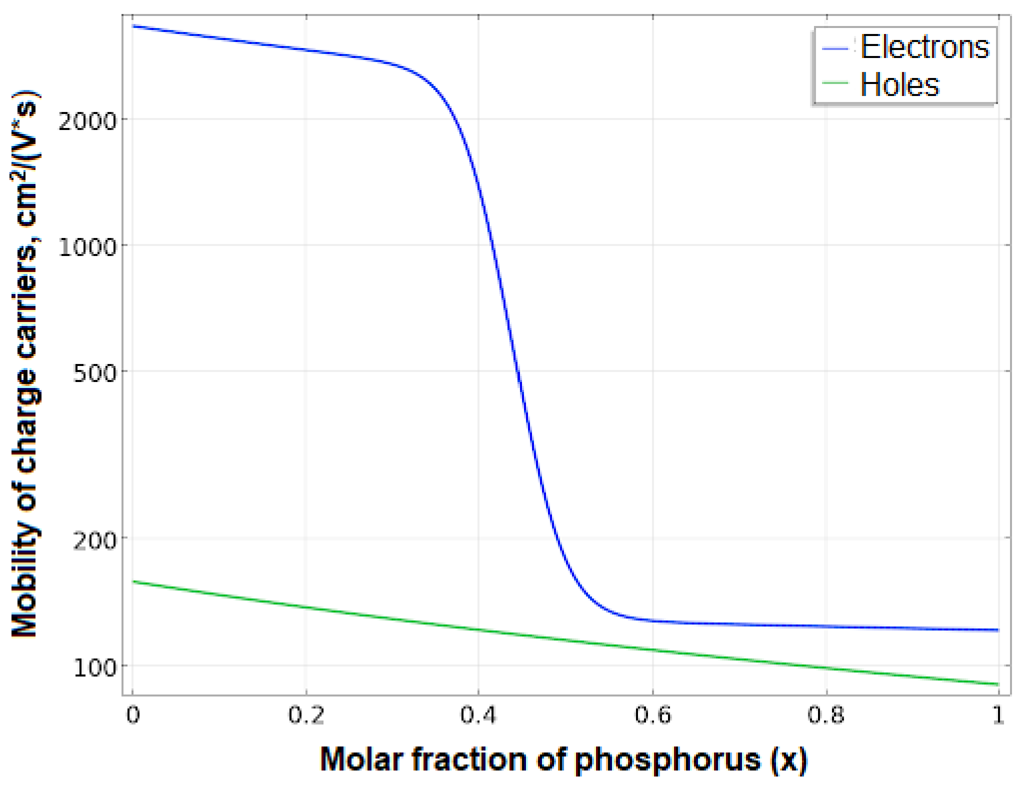

Figure S2. Carrier mobility in GaP_xAs_{1-x} vs. P content x. Doping level is 10^{18} cm^{-3}.

The lifetime associated with the Shockley-Read-Hall (SRH) non-radiative process was calculated using literature data for the electron and hole lifetime of pure GaAs [2,3] and GaP [4], and theoretical data on lifetime, the capture cross-section, and thermal velocity of charge carriers dependency on the material parameters [5]. The minority carrier lifetime in SRH process can be approximately formulated as

$$\frac{1}{\tau} = \sigma \cdot V_{th} \cdot N_t,$$

where τ is the carrier lifetime, σ is the carrier capture cross-section, V_{th} is the carrier thermal velocity, and N_t is the trap density. Herewith, the capture cross-section and thermal velocity depend on material parameters (e.g., carrier effective mass, dielectric susceptibility) via

$$\sigma = \frac{e^4}{36\pi \cdot (\varepsilon\varepsilon_0 kT)^2},$$

and

$$V_{th} = \sqrt{\frac{3kT}{m}},$$

where e is the electron charge, ε is the relative material dielectric susceptibility, ε_0 is the vacuum dielectric susceptibility, k is the Boltzmann constant, T is the carrier temperature, and m is the carrier effective mass.

Thus, the carrier lifetime depends on material parameters, i.e., dielectric susceptibility and effective mass. In our calculations we used a linear approximation of the dielectric susceptibility in the approximation of independent atomic polarization in the compound and using the formerly derived effective mass and state density vs content. Moreover, it is expected that the carriers in different valleys have different lifetimes due to, e.g., different capture cross-sections. In order to account these factors, we used an approach similar to the calculation of the carrier mobility vs content, i.e.,

$$\frac{1}{\tau_e} = \frac{1}{\tau_{GaAs}} \cdot \frac{\varepsilon^2_{GaAs}}{\varepsilon^2(x)} \cdot \frac{V^{\Gamma}_{th}(x)}{V^{\Gamma}_{th\,GaAs}} \cdot F(\Delta E) + \frac{1}{\tau_{GaP}} \cdot \frac{\varepsilon^2_{GaP}}{\varepsilon^2(x)} \cdot \frac{V^{X}_{th}(x)}{V^{X}_{th\,GaP}} \cdot F(-\Delta E),$$

$$\frac{1}{\tau_h} = \frac{1-x}{\tau_{GaAs}} + \frac{x}{\tau_{GaP}}.$$

The derived curves are presented in Figure S3a.

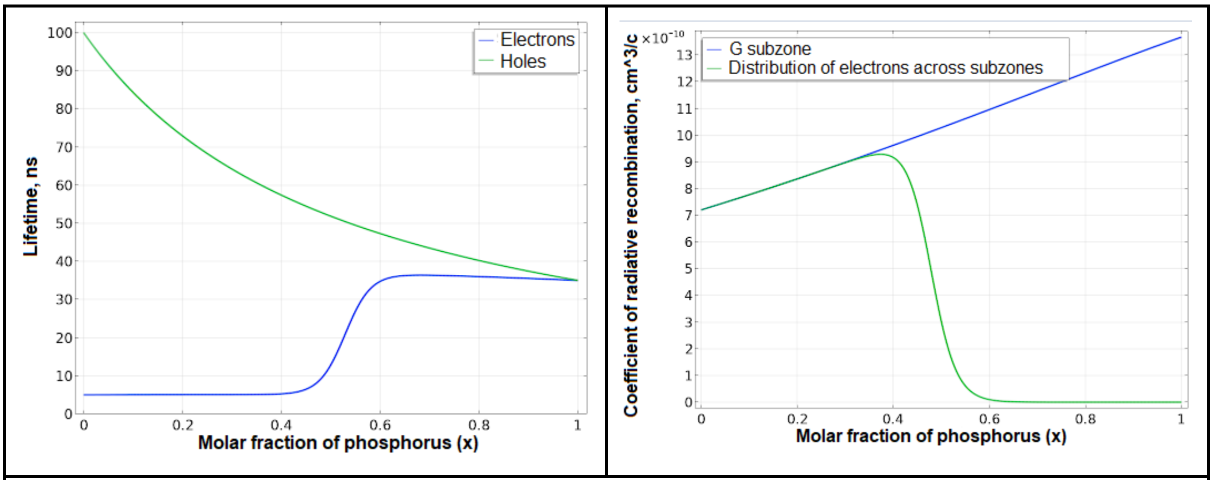

Figure S3. (a) Carrier lifetime in GaP_xAs_{1-x} for SRH recombination process; (b) and the corresponding radiative recombination rate.

The calculation of LED function requires a precise account of the radiative recombination probability. A simple analytical expression can be written for the radiative recombination rate in a bulk material [6]. Based on the analytical expressions in [6], the GaAs recombination coefficient [7], and the previously described effective mass and dielectric susceptibility formulae, we extrapolated the radiative recombination coefficient for the Γ-valley of the given GaP_xAs_{1-x} compound:

$$B(x) = B_{GaAs} \cdot \frac{\sqrt{\varepsilon(x)} \cdot M^{-1}(x) \cdot E_g^2(x) \cdot (m^{-1}_{e-\Gamma}(x)-1)}{\sqrt{\varepsilon_{GaAs}} \cdot M^{-1}_{GaAs}(x) \cdot E^2_{g,GaAs}(x) \cdot (m^{-1}_{e-\Gamma,GaAs}-1)} \cdot F(\Delta E),$$

where the reduced mass is defined by

$$M^{-1}(x) = \frac{(m_e\sqrt{m_{hh}})^{3/2} + (m_e\sqrt{m_{lh}})^{3/2}}{(m_e m_{hh})^{3/2} + (m_e m_{lh})^{3/2}},$$

$B(x)$ is the SRH radiative recombination coefficient, E_g is the direct transition bandgap (in the Γ-subband), m_e is the relative effective electron mass in the Γ-subband, m_{hh} and m_{lh} are the effective heavy and light hole masses, respectively, M is the reduced mass of the radiative transition. The factor $F(\Delta E)$ is needed, as it was in the formulae above, to account for the electron distribution in the subbands. As the electrons move into the X-valley, the material

becomes indirect, and the radiative recombination is suppressed, so the GaP-related can be omitted. The calculated curve is presented in Figure S3b.

The calculations were performed in a framework of 1D model; thus, we did not account for the energy bandgap bending near the NW sidewalls caused by the presence of surface states. However, we suggest that during MBE growth of GaP:Si upper emitters the radial growth of NWs occurred. This should lead to passivation of NWs [8], limiting the influence of these states on charge transport and radiative recombination. The detailed investigation of this effect was out of scope of the current manuscript and will be the subject matter of a following study.

Section 2 Room temperature micro-photoluminescence spectroscopic characterization

Microspectroscopy (μ-PL) study from the as-grown p-i-n-heterostructured GaP/GaPAs NW epitaxial array was performed to confirm the presence of the direct bandgap GaPAs segments in the synthesized NWs. Measurements were carried out at 300K in the backscattering geometry using a Horiba LabRAM HR800 spectrometer equipped with a 600 gr/mm grating, a peltier-cooled Synapse CCD Si-based detector and a x20 NA0.4 Olympus Plan N objective. μ-PL response acquired using the 532 nm laser excitation with an excitation power density of 10^5 W·cm^{-2} is presented in Figure S4. The used objective provides an excitation beam focusing to a spot of ~5 μm diameter on the sample surface, thus the PL signal was collected from the countable number of NWs.

The obtained PL spectrum represents a bright line with intensity maximum at 650 nm and full width at half maximum (FWHM) of 30 nm. Notably, we did not observe any PL response from pure GaP NWs (grown in the same conditions, but without GaPAs insertions). According to Craford et al. [9], the PL emission line at 650 nm corresponds to the composition of zinc blende GaPAs ternary alloy close to $GaP_{0.35}As_{0.65}$ and is consistent with the expected composition for our growth procedure described in [10].

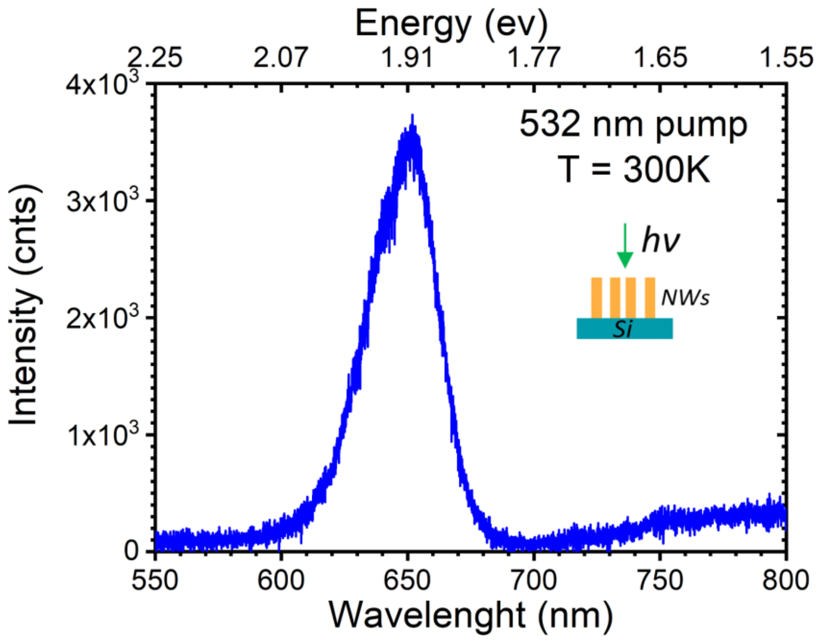

Figure S4. PL spectrum taken from the p-i-n-heterostructured GaP/GaPAs NW epitaxial array.

Section 3 PU synthesis

PU was obtained according to the previously reported procedure [11,12]. Namely, 1.00 g (0.0084 mol) of methyldiethanol amine, 1.41 g (0.0084 mol) of the catalyst (DBTDL) were dissolved in 10 ml of anhydrous DMF under inert atmosphere (Figure S5). The reaction mixture was stirred at 80°C for 24 hours and then cooled down to RT and diluted with 5 ml of DMF. Desired PU was isolated by the precipitation into Et_2O excess and then was thoroughly washed with Et_2O and dried for 8 h at RT and 48 h at 70°C. Yield: 2.31 g (96%); IR: ATR mode, ν, $cm^{-1,}$ w—weak, m—medium, s- strong): 3200 (w, νNH), 3149 (w, νCH_{Alk}), 1625 (s, νC=O), 1552 (s, νC=O), 1463 (m, νCN), 1387 (w), 1305 (w), 1258 (s), 1031 (w, νCO), 789 (w), 630 (w), 596 (w);

N-methyldiethanolamine (>99%, Aldrich), hexamethylene diisocyanate (>98%, Aldrich), dibutyltin dilaurate (DBTDL, 95%, Aldrich), N,N-dimethylformamide (DMF; anhydrous, 99.8%, SeccoSolv), diethyl ether (Et2O, 98%, NevaReactiv) were used without purification. Infrared (IR) spectra were acquired on a Nicolet iS10 FTIR spectrometer in the ATR mode on germanium crystal.

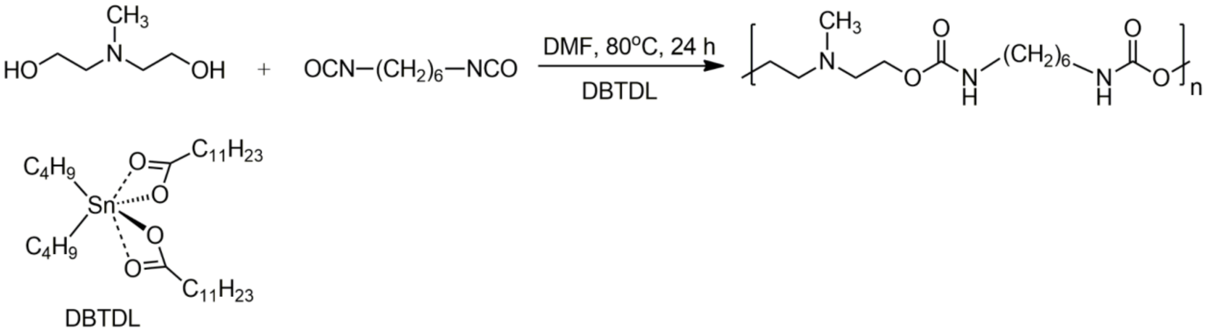

Figure S5. Scheme of PU synthesis.

One of the main advantages of polyurethanes is the ability to vary their mechanical properties in the wide range from high-strength composites to thermoelastomers by choosing the appropriate monomer [12,13]. In this study, we chose linear homopolymers as the substrate owing to the necessary mechanical properties, i. e. rather high strength before break 0.1 GPa and elongation at break more than 800% [11,14]. Due to the aliphatic nature of the selected initial monomers, the polymer had a high solubility in polar solvents such as DMF or dimethylsulfoxide, and due to the linear, rather than cross-linked structure of the polymer itself, the films formed by the solution (casting) method could be removed by dissolving.

However, the optimization of PU dissolution in DMF and the removal of product materials was found to be necessary for efficient PDMS/NW membrane fabrication. The flexible LED devices fabricated using PU cap films will be reported elsewhere.

Supporting Information References

1. Adachi, S. *Properties of Semiconductor Alloys*; John Wiley & Sons, Ltd: Chichester, UK, 2009.

2. Timmons, M.L.; Hutchby, J.A.; Ahrenkiel, R.K.; Dunlavy, D.J. *GaAs and Related Compounds*; Inst. of Phys., Bristol and Philadelphia., 1988.

3. Zarem, H.A.; Lebens, J.A.; Nordstrom, K.B.; Sercel, P.C.; Sanders, S.; Eng, L.E.; Yariv, A.; Vahala, K.J. Effect of Al Mole Fraction on Carrier Diffusion Lengths and Lifetimes in Al x Ga 1− x As. *Appl. Phys. Lett.* **1989**, *55*, 2622–2624.

4. Young, M.L.; Wight, D.R. Concentration Dependence of the Minority Carrier Diffusion Length and Lifetime in GaP. *J. Phys. D. Appl. Phys.* **1974**, *7*, 308.

5. Poklonskii, N.A.; Gorbachuk, N.I.; Syaglo, A.I.; Shpakovskii, S. V. *Investigation of Transition Processes in Semiconductor Structures*; 2009.

6. Asryan, L. V; Suris, R.A. Inhomogeneous Line Broadening and the Threshold Current Density of a Semiconductor Quantum Dot Laser. *Semicond. Sci. Technol.* **1996**, *11*, 554–567.

7. Varshni, Y.P. Band-to-Band Radiative Recombination in Groups IV, VI, and III-V Semiconductors (I). *Phys. status solidi* **1967**, *19*, 459–514.

8. Chang, C.-C.; Chi, C.-Y.; Yao, M.; Huang, N.; Chen, C.-C.; Theiss, J.; Bushmaker, A.W.; LaLumondiere, S.; Yeh, T.-W.; Povinelli, M.L.; et al. Electrical and Optical Characterization of Surface Passivation in GaAs Nanowires. *Nano Lett.* **2012**, *12*, 4484–4489.

9. Craford, M.G.; Shaw, R.W.; Herzog, A.H.; Groves, W.O. Radiative Recombination Mechanisms in GaAsP Diodes with and without Nitrogen Doping. *J. Appl. Phys.* **1972**, *43*, 4075–4083.

10. Bolshakov, A.D.; Fedorov, V. V.; Sibirev, N. V.; Fetisova, M. V.; Moiseev, E.I.;

Kryzhanovskaya, N. V.; Koval, O.Y.; Ubyivovk, E. V.; Mozharov, A.M.; Cirlin, G.E.; et al. Growth and Characterization of GaP/GaPAs Nanowire Heterostructures with Controllable Composition. *Phys. status solidi – Rapid Res. Lett.* **2019**, *13*, 1900350.

11. Morozova, S.M.; Shaplov, A.S.; Lozinskaya, E.I.; Vlasov, P.S.; Sardon, H.; Mecerreyes, D.; Vygodskii, Y.S. Poly(Ionic Liquid)-Based Polyurethanes Having Imidazolium, Ammonium, Morpholinium or Pyrrolidinium Cations. *High Perform. Polym.* **2017**, *29*, 691–703.

12. Morozova, S.M.; Lozinskaya, E.I.; Sardon, H.; Suárez-García, F.; Vlasov, P.S.; Vaudemont, R.; Vygodskii, Y.S.; Shaplov, A.S. Ionic Polyureas—A Novel Subclass of Poly(Ionic Liquid)s for CO2 Capture. *Membranes (Basel).* **2020**, *10*, 240.

13. Akindoyo, J.O.; Beg, M.D.H.; Ghazali, S.; Islam, M.R.; Jeyaratnam, N.; Yuvaraj, A.R. Polyurethane Types, Synthesis and Applications – a Review. *RSC Adv.* **2016**, *6*, 114453–114482.

14. Chan, W.-C.; Chen, S.-A. Polyurethane Ionomers: Effects of Emulsification on Properties of Hexamethylene Diisocyanate-Based Polyether Polyurethane Cationomers. *Polymer (Guildf).* **1988**, *29*, 1995–2001.
